# College of Physicians of Philadelphia, May 6, 1885

**Published:** 1885-06

**Authors:** 


### A CRITICISM OF DR. LEEDS'S PAPER ON "THE COMPOSITION AND METHODS OF ANALYSIS OF HUMAN MILK." *By* A. V. MEIGS, M. D., *Physician to the Pennsylvania Hospital and to the Children's Hospital.*

[Read May 6, 1885.]

*College of Physicians of Philadelphia, stated meeting held May 6, 1885, the President, Dr. J. M. DaCosta, in the chair.*

Denial and reiteration prove nothing. I shall, therefore, endeavor to restrain myself from the use of either of them in my effort to compass my present purpose, which is to persuade the profession of the truth of what I advanced in regard to the composition of human milk, in a paper read before the Philadelphia County Medical Society about a year ago.* I brought forward then certain arguments which seemed to me to prove conclusively that human milk contains only about one per cent. of casein. Those arguments it is not necessary for me to repeat, especially as I have nothing new to advance, and I must therefore refer any one to whom the subject is new,

---

* Proceedings of the Philadelphia County Medical Society, vol. vi., 1883-4.

but who feels an interest in it, to my former paper. At present I wish merely to criticize the results arrived at by Dr. Leeds, and detailed in a paper read before the College.†

In such a criticism I shall not, of course, be restrained by a false delicacy from using all legitimate means to show why we should decline to accept Dr. Leeds's conclusions. The question in dispute is, how much casein human milk contains, and particularly what is its quantity relatively to that contained in cow's milk. Upon the decision of this question must depend the lives of a very large number of infants, for the conclusion at which we arrive as to the relative composition of the two kinds of milk must necessarily decide how we will use cow's milk as a food for young infants.

Dr. Leeds, in his paper, proceeds first to criticise previous methods of analysis and then goes on to describe the method used in obtaining his own results. I shall, therefore, begin by taking up his strictures upon my method of analysis, and then will discuss his own conclusions. In order that his criticism of the method I suggested may be understood, I shall have to give a brief description of my process as originally described. This was first published in the *Philadelphia Medical Times*, July 1, 1882, the paper having been read at the Philadelphia County Medical Society, the previous February.

To carry out the method, 15 c.c. of milk are required. Discharge 5 c.c. of milk from a pipette into a platinum dish and weigh; dry over a water-bath at 100° C. until the weight becomes constant, then incinerate, beginning with moderate heat and finally using the blast flame. This gives the water, total solids, and ash. Discharge 10 c.c. of milk in another dish and weigh, taking care that the weight be exactly double the first

† Transactions of the College of Physicians of Philadelphia, 3d Series, vol. vii., 1884.

amount; pour this into a high narrow 100 c.c. graduated bottle and add 20 c.c. of distilled water, this being used to wash all the milk from the dish. To this add 20 c.c. of ether and tightly stopper the bottle and agitate for five minutes, then add 200 c.c. of alcohol and again agitate for five minutes and let the bottle stand until the fluid separates into two layers. Draw off the upper layer, of ether containing fat, as nearly as can be done without taking any of the lower layer; pour in 5 c.c. of ether to mix with what fat is left, and draw off. Thus 5 c.c. of ether must be poured on and drawn off five times. The ethereal solution being dried over warm, and finally boiling water, gives the amount of fat. There is now left in the bottle the sugar, casein and salts; this must be washed into a dish (best platinum) and dried over the water-bath. The residue is treated with boiling water and then allowed to stand; the casein sinks to the bottom and the sugar remains in solution. The clear solution is poured off and the residue again dried and treated with boiling water, and what is thus obtained added to that already had. This must be repeated until it is found that when boiling water is poured upon the dried sugar, complete solution takes place, no flocculi being seen in it. The casein residue is then, after being thoroughly dried, treated once or twice with boiling water to wash out any sugar that may have been left in it, care being taken that no casein is poured off. This sugar is added to that previously obtained, and the two residues are dried and incinerated, the loss in burning giving the weights of the casein and sugar respectively.

In Dr. Leeds's criticism of my method he finds fault, first, with the use of a pipette in measuring out the portions of milk to be used in analysis, saying that "the milk leaves minute particles upon its walls, and the alteration in composition thus produced is the greater, the more extensive the wetted surfaces of the measuring vessel." This objection, although theoretically correct, is trivial, and the possible error introduced is not nearly so great as that likely to result from his own method of pouring out at a guess 5 c.c. of fluid, and thus have to weigh several times before getting sufficiently near the desired quantity to be able to proceed with the analysis. This is particularly the case where the 10 c.c. for the second portion of the analysis comes to be weighed, for it must weigh exactly twice that first used to make the analysis at all accurate. He says in speaking of the first portion of my method, by which are obtained the water, total solids, and ash—"evaporation to dryness in a water-bath to constant weight, is tedious, usually requiring three hours, and is neither so accurate nor expeditious as the method of coagulation with alcohol," and then after giving a comparison of the results obtained by trial of the two methods says—"in other words, at the expiration of seven hours, I had obtained the same constant weight as I had found by the method of coagulation at the expiration of one and one-half hours." Now he comes to this erroneous conclusion because he makes a mistake in his arithmetic with regard to the results obtained by his experimental trial. The actual fact is, that the addition of alcohol is an improvement in that portion of the analysis, although it expedites the last portion of the drying only very slightly, and does not prevent the formation of a skin upon the surface, as Dr. Leeds states. His mistake is as follows: He says, "to 5.1195 grms. milk added 3 c.c. alcohol, evaporated to dryness on water-bath * * * and then to constant weight in air-bath at 105°. Loss of weight 0.699 grm. or 13.56 per cent." This last sentence contains two mistakes, it should have been—total solids 0.699 grm. or 13.65 per cent. This mistake in the percentage of the total solids is evidently not a mere typographical error, for upon it is based his conclusion that the addition of alcohol so very much expedites the drying. He then proceeds to take a second sample and dry it without the addition of alcohol; he drives off moisture until the total solids left, amounted at the end of seven hours to 13.5 per cent. If he had dried the first sample with which he tested his own method to the same degree as he did the latter, he would have found that it took very little less time, for it is in driving of the last portions of moisture that the process is tedious. The sample dried by his own method was not then, as the figures when corrected show, brought to constant weight, but still contained some moisture, and thus his mistaken conclusion that the addition of alcohol so much expedites the process.

With regard to the extraction of fat, Dr. Leeds says, "When water is present, ether will extract not only fat but substances soluble in water. This was probably the case in the present instance, and experiment confirmed the conjecture. After distilling off the impure ether, drying the fat to constant weight at 105° and weighing, the fat thus obtained was redissolved in absolute ether. In every trial a residue was left behind. This residue dissolved readily in water. It proved to be milk-sugar." I have often tried redissolving the fat residues in absolute ether, and never had any difficulty in causing it all to pass into solution, except in the instances I mentioned in my previous paper, when a white substance was precipitated from the ethereal solutions of fat from human milk. This substance when separated could not be redissolved in ether as I mentioned, and for the moment I thought it must be casein or sugar, but when I found that it melted and looked like any other hot grease the moment it was warmed, I concluded it must be some form of fat for whose reactions and appearance I could not account, nor can I now. The reason Dr. Leeds found milk-sugar with the fat when extracted by my method, must have been that he was careless in pipetting off the ethereal solution of fat, and drew off also some of the water containing milk-sugar, an accident which can only be avoided by observing the greatest care in that portion of the process.

There is little that need be said with regard to the objections brought forward to my method of separating the casein and sugar. Dr. Leeds says casein (albuminoids) is "partly soluble in boiling water." If this is true, my whole method must fall to the ground, but in my paper I stated my conviction, that if casein be once thoroughly hard dried, it entirely ceases to be soluble in water, and this is very easily tested by drying some casein and then trying to redissolve it.

It now becomes necessary to speak of the method of analysis advocated by Dr. Leeds, and of his detail of the work he has done. The method he follows is the Gerber-Ritthausen's, and he says of it, "that it is the only method known at the present time which is precise and rigidly accurate."

This method is described in full in a book entitled *Chemical and Physical Analysis of Milk, Condensed Milk, Infants' Food, etc.*, by Dr. Nicholas Gerber, translated by Dr. Hermann Endemann, New York, 1882. The method, as described by Dr. Leeds, is as follows:

"*Totals Solids.*—Weigh off 5 grms. of milk in a tared covered platinum capsule. Coagulate with absolute alcohol (about 3 c.c. are used), and evaporate to dryness on water-bath. Transfer to drying-oven, and keep at 105° C. until constant weight is attained.

"*Ash.*—Ignite the residue first over a small flame, and finally at a dull-red heat. Cover the dish, cool in the desiccator and weigh.

"*Albuminoids.*—Dissolve 63.5 grms. pure sulphate of copper in a litre of water. Prepare also a potash solution containing 50 grms. caustic potash in 1 litre. Weigh out 10 grms. of milk in a covered beaker glass, and dilute with 100 c.c. water. Add 2.5 to 3 c.c. of the copper solution. Then run in sufficient potash to neutralize exactly the excess of sulphate, which will require about 1.25 to 1.5 c.c. of the potash. The coagulated albuminoids settle immediately, leaving the liquid clear. In testing the reaction, the stirring-rod, which has been washed and withdrawn from the solution as soon as the potash has been stirred in, is dipped into the clear supernatant liquid. A drop of this liquid should turn neutral test-paper neither blue nor red. Care should be exercised not to allow the stirring-rod to bring up particles of the coagulum, since these interfere with the reaction. The clear liquid is then decanted through a filter-paper, previously dried at 110°, and weighed in a weighing flask. The precipitate is then stirred up with 100 c.c. water, allowed to settle, the supernatant liquid again decanted through the filter, and, finally, the precipitate is washed upon it. The beaker is thoroughly cleansed with a rubber washer; and all these filtrates, amounting to about 240 c.c., are finally made up to exactly 250 c.c. for the determination of milk-sugar.

"The filter-paper containing the precipitate is then opened out upon a large watch-glass, and, after drying to a certain point, is divided up into small particles by a platinum spatula, and this comminution is repeated from time to time, until finally the whole mass becomes a fine powder.

"*Fat*—The filter-paper containing the precipitate is gathered up and placed loosely in a proper funnel. The beaker-glass used for the precipitation is washed out with ether to dissolve any traces of fat adhering to it, and these ethereal washings are poured through the funnel and allowed to run into a small weighed flask, with which the funnel is connected by a groundglass joint. The funnel is then connected with a return-cooler, the flask carefully heated by a water-bath, and the filter paper is made to swim in the ether condensed in the funnel for about an hour, when the extraction of fat will be complete. The ether is distilled off, the flask dried at a temperature of 105°, cooled in a desiccator, and weighed. Its increment in weight gives the amount of fat.

"*Albuminoids.*—The residue in the filter is dried at 110°, and weighed in the weighing flask until constant weight is attained. It is then ignited in a platinum crucible, and the weight of ash deducted. The loss of weight is the amount of albuminoids.

"*Milk Sugar.*—This is determined in the filtrate by Fehling's solution. The figures thus obtained are identical with those found by evaporation of the filtrate to dryness, igniting, and substracting ash."

The first and most important objection to this process is that there are added to the milk to be analyzed fixed substances, which are believed to form new compounds with some of its constituents. Thus, Dr. Leeds tells us, that an albuminate of copper is formed and precipitated. In his criticism of Haidlen's method he adds a foot-note, saying: "The addition of gypsum, marble, glass, sand, etc., is unnecessary, and a source of error." Why is not the addition of potash and sulphate of copper a much greater source of error? The first mentioned substances are comparatively inert, and little disposed to form new combinations, while the latter are among the most active in the strength of their chemical affinities. No method of analysis can be accepted as conclusively accurate which depends upon the introduction of a reagent which forms a new compound which is insusceptible of being again separated into its original parts, to show how much of each is present when the analysis is completed. How is it possible to know that the incineration of any given weight of albuminate of copper, formed from human milk, which has been dried at 100° or 105°, will inform us how much casein was originally needed to make up the compound, when it is remembered that casein itself is a substance about which we still know so little that it is not yet positively certain whether it is simple or compound?

Dr. Leeds's reagents for precipitating the albuminoids are almost exactly those used to make the Fehling's copper-test. Why, therefore, is it not likely that some of the sugar is precipitated or altered by them, as would surely happen if the fluid was heated, thereby preventing its reacting to the Fehling's solution, with which, as I understand it, Dr. Leeds made his test of the purity of his supposed casein residue. Dr. Leeds himself suggests another objection, "that, in the precipitation of the albuminoids by Ritthausen's solution, hydrated basic sulphate of copper is precipitated." But, he says, this objection does not hold good, as he has "failed to detect it in the presence of more than traces of hydrated basic sulphate." For my own part, I made the experiment of mixing the reagents, as directed, in water simply, without the presence of milk. So soon as the copper and potash solutions were brought together, a precipitation of course took place. This precipitated I collected and dried at 100°, and then exposed to the heat of a gas flame. The difference between the weight of the precipitate dried in a water-bath at 100°, until it ceased to lose weight, and after it had been exposed to the heat of a flame, was (the same bulks of the reagents being used as directed for analysis) 0.038 gramme. This makes, if the same thing occurs when the method is used for analytic purposes, the casein too great in quantity by about 0.35 per cent., which is nearly the difference between Dr. Leeds's conclusion and my own as to the quantity of casein in human milk. Although the conditions are different, when the reagents alone are used, from those where they are used in analysis, and it cannot be argued that there must necessarily be a too high rating of the casein from this cause, yet, on the other hand, it can be by no means proved that such an error does not result; and, of the two possibilities, the latter seems much the more probable. This matter alone, even if there was no other objection to the process advocated by Dr. Leeds, is sufficient reason for declaring the method absolutely unreliable in the present state of knowledge of the subject.

I made experimental trials of the Gerber-Ritthausen's method, which Dr. Leeds declares to be the only accurate one known. The details of these trials it is hardly necessary to describe here; the result was, that the Gerber-Ritthausen's method rated the fat and milk-sugar a trifle lower, and the casein higher, than my own method did when applied to the same milk. The ash and water came out the same by both processes. With regard to the ash I found that it made no difference whether the incineration was done with an ordinary flame or with the blast, or whether in a closed or open vessel. If the burning was thoroughly done with the ordinary flame, the application afterwards of the blast did not drive off anything more; nor was there any difference in the weight of the residue, whether the vessel was open or covered. With regard to the fact that Dr. Leeds's process gave a slightly less amount of at than did my own, although the difference was so slight as to be hardly worth mentioning, it struck me that, perhaps, the potash which is used may react to a slight degree upon some of the fat, and carry it down into some new form with the casein, which is acted upon by the copper. This is only a further reason for declining to accept conclusions deduced from a process which is open to so many sources of error, no one of which can be really proved to be inoperative.

The next matter I have to take up in connection with the work Dr. Leeds has done in milk analysis, is one which I approach with a good deal of hesitation, nor would I mention it at all in a spirit of fault-finding criticism, but only because it is necessary to have an exact appreciation of the correctness of his work, in order that a just estimate of its value may be attained. The table which he gives and which embodies the results of his analyses of eighty samples of human milk, has in it more errors than should exist when deductions are drawn intended to convince scientific men of the correctness of an estimate of the composition of human milk which, to the present time certainly, has not received anything like universal, or even general acceptance. I have carefully gone over this table and find that in seven out of his sixty-four separate analyses, the figures in the column headed "total solids by addition of constituents" are incorrect. The errors are all small except in analysis No. 32, but in it the error is quite a large one. The errors in Nos. 24, 32, 46, 50, 54, 56–59, and 75–80 (marked, "anæmic six cases"), Dr. Leeds gives at the the end of his table in parallel columns the maximum, minimum, and average of each of the different constituents. In the average column the figures given are all incorrect. It is not easy to say exactly how these averages were arrived at, for Dr. Leeds (page 243 loc. cit.) says that in estimating the "maximum difference" he omitted "Laboratory No. 1063" as being affected by some accidental error, and used sixty-two separate analyses, which would seem to leave out also the last two analyses, which are marked respectively, "robust six cases" and "anæmic six cases." My table shows in parallel columns Dr. Leeds estimate of the average and corrected figures deduced in different ways.

| | Leeds's Average. | Average obtained by including all the analyses. Estimating Nos. 56-59 and 60-63 as 4 each, and the last two (Nos. 69 80) as 12. Dividing all the sums, except ash and total solids directly by evaporation, by 76, and those by 80. | Analysis No. 1063 omitted, otherwise same as previous estimate, but the sums divided by 75 and 79 respectively. | Analysis No. 1063 and the last two omitted, consequently the sums divided by 57 and 61. |
|---|---|---|---|---|
| I. Specific gravity | 1.0313 | ---- | ---- | ---- |
| II. Albuminoids | 1.995 | 1.949 | 1.910 | 2.139 |
| III. Sugar | 6.936 | 6.954 | 6.975 | 7.738 |
| IV. Fat | 4.131 | 4.127 | 4.137 | 4.637 |
| V. Solids not fat | 9.137 | ---- | ---- | ---- |
| VI. Ash | 0.201 | 0.218 | 0.219 | 0.237 |
| VII. Total solids (by addition of constituents) | 13.268 | 13.253 | 13.245 | 14.750 |
| VIII. Total Solids (directly by evaporation) | 13.267 | 13.237 | 13.235 | 14.637 |
| IX. Difference between VII and VIII | 0.001 | 0.016 | 0.010 | 0.113 |
| X. Water | 86.732 | ---- | ---- | ---- |

Dr. Leeds says : " Thus, it will be seen from the accompanying table, giving the results of sixty-two separate analyses of human milk (excluding Laboratory No. 1063, as being manifestly affected by some accidental error), the maximum difference is 0.21 per cent.

" The average error, as determined by ordinary arithmetical methods, is 0.001 per cent."

A glance at the table shows how far from the actual facts this statement is ; for, if the first sixty-two analyses only are considered, and No. 1063 omitted, the error, instead of being 0.001 per cent., is 0.113 per cent., or just one hundred and thirteen times greater than is stated. The errors are all, it is but just to state, small, still the existence of so very many of them must necessarily cast much doubt upon the value of the whole work, especially when it is considered how very easily many of them could have been avoided, as, for instance, in the matter of the making up of the column "total solids, by addition of constituents," when the sum in addition is so very simple a one.

I may sum up by saying that Dr. Leeds's conclusions must not be accepted, for the following reasons:

First, because he has failed to disprove any of the propositions I put forth in my previous paper, having brought no proof to bear substantiating his assertion, that the Gerber-Ritthausen's is "the only method known at the present time which is precise and rigidly accurate."

Second, that the reasons given for estimating the casein amount low (about 1 per cent.), still hold, in the absence of disproof.

Third, that the process advocated by Dr. Leeds is necessarily unreliable, because there are added extraneous fixed substances which form new compounds with the milk, rendering it impossible to make accurate determinations of the various constituents.

Fourth, because of the numerous and considerable inaccuracies which I have pointed out in the paper.

After the reading of the preceding paper:—

Dr. ALBERT R. LEEDS, said: I shall refer briefly to some of the objections made by Dr. Meigs. The plan taken by Dr. Meigs of critizing the minutiæ of the work is an entirely just one, inasmuch as we propose to arrive at results of as great scientific accuracy as possible.

In regard to the first point that he speaks of. It is a little point to be sure, but he is certainly not correct in stating that it makes no difference whether we draw off milk for analysis with a pipette or pour directly into a dish. Milk is not a homogeneous liquid in the sense that it does not contain minute particles which have the power of attaching themselves to the walls of the vessels, and the constitution of the milk after it has left the pipette is somewhat altered by the leaving behind on the walls of the pipette of some of these minute particles.

2d. In regard to the propriety of measuring a portion as compared with weighing a portion. There can be no doubt in the mind of any chemist as to which is the more correct. The only method which can lay claim to accuracy is that by direct weighing. He speaks as though it were a matter of difficulty to obtain an accurate weight. Such is not the case. When supplied with a beaker, which has its own tare or weight marked on the glass, a little experience enables one to estimate how much is to be poured in to make a definite weight of milk. The beaker is kept covered during weighing to prevent evaporation. When a measured quantity is employed, we cannot arrive at definite results until the specific gravity is ascertained. This method is so unsatisfactory that no chemist thinks of making a determination of milk analysis upon a measured quantity, but always upon a weighed sample. If, in a legal case relating to the adulteration of milk, it were discovered that the chemist had used measures instead of weight, I think that his testimony would be thrown out at once.

3d. As to the amount of time consumed in evaporation. A great many experiments have been made upon the relative thoroughness of drying by means of alcohol and drying without alcohol. The results are identical, provided one does evaporate to a constant weight. As a curious matter bearing on this point, I may say that my results upon the determination of total solids were checked off by other chemists on the State Board of Health, who used other methods of drying. This seems to be a small matter, but inasmuch as the State law requires that in the State of New Jersey, cow's milk shall contain above 12 per cent. of total solids, the method of drying becomes of great importance. It was found that the results were practically identical, the only difference being that by the addition of alcohol a great deal of economy of time was attained.

4th. In regard to the use of ether as a solvent for the fat in the manner proposed by Dr. Meigs. I fail to see that there can be any justification whatever for its use in this way. Whenever it is necessary to extract fat from an organic body, the universal rule is, that even in a food substance like flour or meal, which contain very little moisture, previous drying of the organic substance should be resorted to before extraction of the fat with ether, because chemists regard the presence of so minute an amount of water as is to be found in food substances, as sufficient to hydrate the ether sufficiently to carry into solution other matters. This is as thoroughly verified as any point in chemistry. I look with perfect astonishment upon a method of fat extraction which proposes to add 10 c.c. of ether to milk containing already as much as 37 per cent. of water to begin with, and then add two or three volumes of water in addition. You get very little ether and an enormous amount of water, and as a result other materials are necessarily extracted. It is not for me to go into what other analyses have been performed. I only speak of the analyses which I have performed myself, and I found that the water had extracted other substances, and I should have thought it very remarkable if it had not done so.

5th. As regards the addition of foreign substances. In the early days of chemistry, at the very beginning of analytical work, it was considered that the bodies themselves should be actually separated by means of crystallization, different solubilities, and so on. These methods are now largely given up as lacking in accuracy. On the contrary, the great point in analytical determination now is to obtain some compound of the body which we desire to isolate, this compound being one of very definite composition, and capable of complete separation from the other substances with which it is associated. The objection has been made to the addition of subtances like copper, and to the addition of substances like sand, glass, gypsum, and so on. The objection to the addition of these latter substances rests on an entirely different ground from that which applies to the addition of copper. When you add any of these latter substances no chemical union takes place, and the residue which is left behind after evaporation is highly hygroscopic. As direct evaporation gives all that is desired, they should not be used.

The addition of copper is resorted to for an entirely different object. It is added because it forms a most insoluble compound with the albuminoids and a compound which is capable of being manipulated, filtered, and washed with thoroughness. You may form an albuminate of zinc, of iron, or of other substances, but none of them, as has been shown by Rithausen, who has made a more extensive study of the albuminoids than any other observer, possesses the facility for manipulating and for thorough washing as the albuminate of copper.

The action of potash on casein could only happen in crude manipulations. In the manipulation of these albuminoids one obtains in the first place, in an acid solution, a precipitate of albuminate of copper; he then adds potash, not to such a point as to act on the albuminate, but to such a point as will just take up the sulphuric acid removed from the copper. It is added to the point of exact neutrality and in the presence of a strong mineral acid like sulphuric. I think that there is not the slightest danger of the potash dissolving the casein. Unquestionably it would do so if added to excess.

Then as to the albuminate of copper itself, the experiment detailed was of entirely different description. On the addition of caustic soda to the copper solution, a precipitate of hydrate of copper would be obtained, which by boiling alone would give up its water and be converted into the oxide of copper. The experiment is not at all analogous to the changes occurring in the manipulation of albuminate of copper. When the albuminate of copper is obtained, it is washed thoroughly, dried, and then ignited, thus finding the exact weight of the mineral portion. This subtracted from the previous weight gives the weight of the organic constituents. I do not see that anything is lacking to make this mode of determining the albumenoids an accurate method. The presence of the basic sulphate of copper in the precipitated albuminate I have been unable to verify with certainty. The reduction of the copper in Fehling's test is a matter of an entirely different description. This is dependent upon the formation of a sub-oxide of copper. There is no trace of reduced copper when albuminate of copper is precipitated as proposed in the analysis of milk, and hence there can be no reduction of any substance of the nature of milk sugar.

In regard to the small errors in addition, this is a matter of entire news to me. I am much surprised to learn that such is the case, but as to the conclusions being shaken, that I cannot suppose there is the slightest ground for believing on the strength of the evidence afforded to-night. The accumulation of testimony has been wholly adverse to the conclusions obtained by Dr. Meigs. They are unsupported by any other chemists who have worked on this subject. So far as I know, he is the only one who has advocated his view as to the small percentage of casein which he supposes to be present in woman's milk.

DR. MEIGS: I merely wish to reply to one thing. The remarks of Dr. Leeds would seem to show that I had taken a measured instead of a weighed quantity. I always weigh the quantity. I use the measure simply to get at the neighborhood of the weight. I use the pipette just as Dr. Leeds uses the beaker glass.

